# Classification of target tissues of *Eisenia fetida* using sequential multimodal chemical analysis and machine learning

**DOI:** 10.1007/s00418-021-02037-1

**Published:** 2021-11-08

**Authors:** Sven Ritschar, Elisabeth Schirmer, Benedikt Hufnagl, Martin G. J. Löder, Andreas Römpp, Christian Laforsch

**Affiliations:** 1 Department of Animal Ecology i and BayCEER, University of Bayreuth, Bayreuth, Germany; 2 Department of Bioanalytical Sciences and Food Analysis, University of Bayreuth, Bayreuth, Germany; 3 Institute of Chemical Technologies and Analytics, Vienna, TU Austria; 4 Purency GmbH, Walfischgasse 8/34, T1010 Vienna, Austria

**Keywords:** Multimodal imaging, *Eisenia fetida*, Random decision forest, Tissue analysis, MALDI-MSI, FTIR

## Abstract

Acquiring comprehensive knowledge about the uptake of pollutants, impact on tissue integrity and the effects at the molecular level in organisms is of increasing interest due to the environmental exposure to numerous contaminants. The analysis of tissues can be performed by histological examination, which is still time-consuming and restricted to target-specific staining methods. The histological approaches can be complemented with chemical imaging analysis. Chemical imaging of tissue sections is typically performed using a single imaging approach. However, for toxicological testing of environmental pollutants, a multimodal approach combined with improved data acquisition and evaluation is desirable, since it may allow for more rapid tissue characterization and give further information on ecotoxicological effects at the tissue level*.* Therefore, using the soil model organism *Eisenia fetida* as a model*,* we developed a sequential workflow combining Fourier transform infrared spectroscopy (FTIR) and matrix-assisted laser desorption/ionization mass spectrometry imaging (MALDI-MSI) for chemical analysis of the same tissue sections. Data analysis of the FTIR spectra via random decision forest (RDF) classification enabled the rapid identification of target tissues (e.g., digestive tissue), which are relevant from an ecotoxicological point of view. MALDI imaging analysis provided specific lipid species which are sensitive to metabolic changes and environmental stressors. Taken together, our approach provides a fast and reproducible workflow for label-free histochemical tissue analyses in *E. fetida*, which can be applied to other model organisms as well*.*

**Supplementary Information:**

The online version contains supplementary material available at 10.1007/s00418-021-02037-1.

## Introduction

Environmental toxicology describes the research on impacts and fate of chemicals introduced into the environment (Leblanc 2004). Such research avails itself of various model organisms with which toxicological experiments are conducted. Prominent examples are the aquatic model *Danio rerio* (zebrafish) (Nagel 2002) or earthworms such as *Eisenia fetida* as terrestrial models (Spurgeon et al. 2003; Molnár et al. 2012; Nayak et al. 2018; Tirado-Ballestas et al. 2020). The impact of toxicants, such as heavy metals (Ali et al. 2020), herbicides or pesticides (Belsky and Joshi 2020), is thereby investigated on different levels. For instance, for earthworms, parameters such as body weight (Zhou et al. 2013), immune system response (Alves et al. 2019), gene expression (Alves et al. 2019) or histological examination (Li et al. 2020) are utilized. Histological assessment, for example, is frequently used for investigation of lesions or malformations of the digestive system of earthworms after exposing these organisms to toxicants (Rodriguez-Seijo et al. 2017; Nayak et al. 2018). This type of analysis requires a time-consuming embedding of conserved specimen in paraffin and thin sectioning using microtomes followed by staining and optical examination under a microscope (Dempster 1963; Sanderson et al. 1988).

In recent times, histological analysis has been enhanced by chemical imaging analysis, which adds a different perspective to the analyzed tissue sections. Chemical imaging enables a detailed analysis, shedding light on the impact of pollutants at a high spatial resolution. In this context, various spectroscopic methods, including Fourier transform infrared spectroscopy (FTIR) (Giorgini et al. 2018) or mass spectrometry (MS) imaging (Liebeke et al. 2015), are applied.

FTIR is a state-of-the-art vibrational spectroscopic method (Diem 2015), which is frequently utilized for the noninvasive analysis of cells (Kimber et al. 2016) or imaging of tissues for diagnosis of inflammatory processes (Movasaghi et al. 2008; Rodrigues et al. 2018). It provides a chemical fingerprint of the analyzed sample, enabling the classification of tissues (Wood et al. 2006) for diagnosis, for example benign versus malignant (Baker et al. 2014). Through correlation of molecular signatures with histological features of an analyzed tissue section, it further allows for characterization of different tissue types (Großerueschkamp et al. 2015). This may be supported by algorithms such as machine learning (Nguyen et al. 2021), which provide the possibility for fast and reproducible analysis of the spectral data (Antora et al. 2019; Kedzierski et al. 2019).

Matrix-assisted laser desorption/ionization mass spectrometry (MALDI-MS) imaging allows for the analysis of proteins (Cazares et al. 2009; Huber et al. 2018), neuropeptides (Chen et al. 2009), lipids (Niehoff et al. 2014) and small molecules, even up to single-cell resolution (Römpp et al. 2010; Schober et al. 2012). MS imaging promotes an understanding of the molecular processes in different samples (Cornett et al. 2007) and has already been used, for example, for mapping and imaging of lipids in sections of whole organisms (Niehoff et al. 2014; Khalil et al. 2015). MS imaging has garnered increasing interest in studies on the effects of environmental pollution in different organisms. For example, zebrafish (*D. rerio*) were used to evaluate the impact of the insecticide fipronil, which mainly affected the eyes of the animals by disturbing the phospholipid metabolism (Liu et al. 2020). In addition to aquatic organisms, rodent model organisms were used to demonstrate the adverse impact of the environmental pollutant bisphenol S on the kidney (Zhao et al. 2018). Zhang et al. (2020) also showed the application of MS imaging for the analysis of the effect of graphene nanoparticles at the metabolic level in *E. fetida*.

The impacts of pollutants on *E. fetida* are commonly assessed on a histological level via common histochemistry using target-specific staining methods (Lapied et al. 2011; Molnár et al. 2012; Wang et al. 2015; Jiang et al. 2020). The application of both described methods in a multimodal workflow connecting a fast fingerprinting approach (FTIR imaging) with molecular specificity (MS imaging) may facilitate the analysis of biochemical changes in distinct tissue types. However, a sequential multimodal approach combined with improved data acquisition and evaluation allowing for more rapid tissue characterization has not been applied for these soil model organisms.

Hence, we developed a sequential multimodal imaging approach combining FTIR and MS imaging on the same tissue sections of the ecotoxicological model organism *E. fetida*. FTIR and subsequent data analysis based on random forest classifiers was used for rapid tissue type identification. MALDI-MSI was applied for the analysis of lipids, as the various functions of lipids, for example in tissue integrity as membrane lipids (Dowhan and Bogdanov 2002) or storage (Welte and Gould 2017), renders them suitable markers for biological changes in earthworms in response to pollutants (summarized in (Solé 2020).

## Methods

### Cryosectioning of *E. fetida*

The *E. fetida* specimens and coconut fiber substrate were purchased from a local provider (https://www.wurmwelten.de). The animals were held in a climate chamber at a constant temperature of 25 °C ± 1.5 °C, humidity of 70% ± 5% and artificial day-night rhythm (12 h light/12 h darkness). The substrate was additionally spiked with oatmeal. For cryosectioning, adult worms were isolated and sedated with 7% magnesium chloride hexahydrate (Merck KGaA, Darmstadt, Germany) to prevent contraction of the digestive organs. Sedated worms were quick-frozen with liquid nitrogen and then placed on a brass plate, cooled with dry ice. Then they were cut, and segments of the individuals were embedded in 3% sodium-carboxymethylcellulose (CMC) (Merck KGaA, Darmstadt, Germany) and frozen on dry ice. Embedded specimens were stored at −80 °C until cryosectioning. The preparation of the sections was performed using a Leica cryostat (Leica CM 1950, Leica Biosystems, Wetzlar, Germany) at a temperature of −19 °C and with carbon steel microtome blades (Feather C35 microtome blades, pfm medical AG, Cologne, Germany). Sections used for analysis were produced at a thickness of 20 µm. The sections for our workflow application were placed on calcium fluoride windows (⌀ 25 mm × 2 mm, Korth-Kristalle GmbH, Kiel, Germany). Reference sections that were used for staining or MS imaging only were placed on Superfrost glass slides (Carl Roth GmbH + Co. KG., Karlsruhe, Germany). Sections that were not immediately processed were stored at −80 °C. Prior to FTIR and MALDI-MSI measurements, sections were transferred directly from the −80 °C storage into a desiccator for 30 min.

### FTIR imaging

FTIR imaging was applied because it preserves tissue integrity while still enabling us to define regions of interest for subsequent MS imaging, which cannot be performed after classical histological staining such as hematoxylin and eosin (H&E).

The FTIR images were acquired using a focal plane array (FPA) detector-based micro-FTIR spectrometer: FTIR spectra of the images were recorded using a ×3.5 IR objective on a Bruker HYPERION 3000 FTIR microscope (Bruker Corporation, Billerica, MA, USA) coupled to a TENSOR 27 spectrometer. The FTIR measurement was conducted in transmission mode on the calcium fluoride windows in a wavenumber range of 3600–900 cm^−1^ with a resolution of 8 cm^−1^ and co-addition of 32 scans. Background measurement on the calcium fluoride window with a pure CMC layer was conducted with the same parameters. FTIR spectra were recorded with a liquid-nitrogen cooled 64 × 64 detector pixel FPA resulting in a spatial resolution of 11.05 µm per pixel. Measurements were obtained with Bruker OPUS software version 7.5 (Bruker Corporation, Billerica, MA, USA).

### Data processing and analysis for FTIR measurements

Analysis of the generated FTIR data was performed using Epina ImageLab version 4.1 (EPINA GmbH). For the separation of tissue types within the cross sections of *E. fetida*, a model based on random decision forest (RDF) classification was established (Breiman 2001). First, tissue types were selected. Four tissue type classes were defined as follows: (1) background, consisting of data within the hyperspectral image that described the embedding medium and the CaF_2_ window; (2) body wall, consisting of data within the hyperspectral image that described the circular and longitudinal muscles, the epidermis and the cuticle; (3) digestive system, consisting of data within the hyperspectral image that described tissue types belonging to the whole digestive system (e.g., stomach and gut); and (4) other tissue, consisting of data within the hyperspectral image describing tissue and information that was not assigned to classes 2 or 3 (e.g., coelomic fluids or chloragogenous tissue). For training of the model, five independent tissue sections of different worms were imaged and used for the preparation of five training data sets. For every tissue section, 30 data points (spectra) for each of the four classes were collected, resulting in a total data collection of 120 data points per training data set (overall total 600 data points). Using spectral descriptors (Hufnagl et al. 2019) in combination with iteratively reducing the number of variables based on the variable importance measure (Breiman 2001), the dimensionality of the data set was reduced from 700 to 215 descriptive variables. Test application was performed on independent tissue sections of *E. fetida* from different individuals. Finally, the performance of the model was validated with a statistical performance assessment.

### Random forest statistical performance assessment

The classification performance of the RDF classifier was assessed by means of Monte Carlo cross-validation (Xu and Liang 2001), which is also known as random sub-sampling validation (Westad and Marini 2015). Cross-validation is a broadly applied methodology for evaluating machine learning models. It is applicable to both classification and regression tasks and comes with the advantage that no separate test data set is required.

In a Monte Carlo cross-validation experiment, the data set of reference spectra is split randomly into a training and a test data set following a certain splitting ratio. The training data is used for training an RDF model which is then used to predict the labels of the test data set. The process is repeated multiple times, where each random split creates a new training/test data set pair.

By collecting the number of correct and wrong predictions for each training/test data set pair, a confusion matrix can be constructed which is shown in Fig. S1. The entries of the main diagonal represent the correctly classified cases, whereas the off-diagonal elements are the wrongly classified cases. In our case a splitting ratio of 0.9 was used to create 20 training/test data set pairs where 90% of the references are used for deriving an RDF model whereas 10% are used for testing. Class-specific performance measures (Ballabio et al. 2018) are listed in Table S1. The overall classification performance measures of total accuracy, Cohen’s kappa and the extended Matthew correlation coefficient were computed as 0.9292, 0.9056 and 0.9064, respectively (Table S1). Accuracy corresponds to the ratio of correct classifications over the total number of samples. Though this measure is one of the most commonly used, it depends on the relative class sizes (meaning the number of training data in each class). Cohen’s kappa, on the other hand, compensates this issue. Consequently, the total accuracy is higher than Cohen's kappa as it is biased towards the performance of the larger classes. The extended Matthew correlation coefficient is another multi-class performance measure commonly used in chemometric studies. More detailed definitions of classification performance measures can be found in the paper by Ballabio et al. (2018) including MATLAB scripts for computing these measures.

### Matrix application

Matrix application for MS measurements was carried out using a semi-automatic pneumatic sprayer system. All sections were coated with 4-nitroanilin matrix (pNA, ≥ 99%, Sigma Aldrich Chemie, Taufkirchen, Germany) at 5 mg/mL in 3:1 acetone/water. We chose pNA as a matrix for our measurement experiments since it is proven to be advantageous for lipid analysis (Steven et al. 2013). On-tissue MS/MS experiments were performed to confirm the identification of lipids showing a spatial distribution within targeted tissue classes. Afterwards, data analysis combining our results of the FTIR and MALDI MSI measurements was performed.

### MS imaging

MALDI-MSI measurements were performed on a Q Exactive™ HF Hybrid Quadrupole-Orbitrap mass spectrometer (Thermo Fisher Scientific, Bremen, Germany), coupled to the atmospheric pressure MALDI imaging source AP-SMALDI10 (TransMIT GmbH, Gießen, Germany). The ion source is equipped with a *λ* = 337 nm N_2_ laser operating at a repetition rate of 60 Hz. Measurements were carried out in positive ion mode with one scanning event and 30 shots per pixel at a mass resolution of 240 k @ *m*/*z* 200 full width at half maximum (FWHM). All measurements were performed with a fixed C-trap injection time of 500 ms. Step sizes were set to 5 µm. Tentative identification of lipids from *E. fetida* sections was based on an online database search (Sud et al. 2007) and on tissue MS/MS of lipids with a precursor isolation window width of ±0.2 *m*/*z*.

### Data analysis for MS imaging

Conversion of proprietary Thermo RAW files to imzML was performed using the Java-based open-access jimzMLConverter software (Race et al. 2012). Ion images and RGB composite images were generated in MSiReader version 1.0. Images were generated using a bin width of ±2.5 ppm. Mass deviations across imaging data sets are given as the root mean square error (RMSE) of the ∆m values in ppm of each individual spectrum containing the targeted ion within a window of ±4 ppm of the exact mass.

### Histological staining

H&E staining was performed after the application of the multimodal imaging approach for histological comparison with the MS imaging results. H&E staining was used to retrospectively evaluate the results of FTIR and MS imaging during the establishment of the workflow, e.g., training of the model for the FTIR. Once validated, the FTIR analysis enables fast identification of regions of interest while preserving the tissue integrity of the tissue sections for subsequent MS imaging analysis (which is not compatible with H&E-stained tissue sections).

Prior to H&E staining, the previously applied matrix for MALDI-MSI needs to be removed without excessive destruction of the tissue section. For matrix removal, acetonitrile has proven to be an adequate solvent. After matrix removal, sections were rinsed in tap water and then stained. For H&E staining, Mayer's hemalum solution acid (Carl Roth GmbH + Co. KG., Karlsruhe, Germany) and 0.1% eosin G (Carl Roth GmbH + Co. KG., Karlsruhe, Germany) were used. Samples were stained for 10 min with hemalum solution (Carl Roth GmbH + Co. KG., Karlsruhe, Germany), washed in tap water for 10 min and then counterstained with 0.1% eosin G for 2 min, followed by dehydration with consecutive increasing ethanol concentrations, isopropanol, xylene, and mounted with Eukitt (Kindler GmbH, Freiburg, Germany). Pictures were acquired with a digital microscope (Leica DVM6, Leica Biosystems) and LAS X version 3.0.8 software (Leica Biosystems).

## Results and discussion

**Fig. 1:**
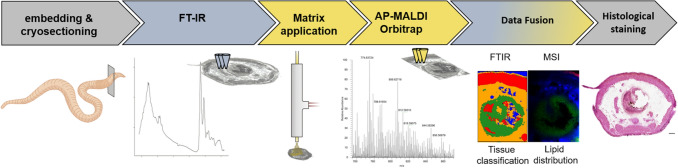
Visualization of the schematic workflow for the multimodal imaging of *E. fetida*. Scale bar = 250 µm

Our study presents an analytical workflow for the analysis of tissue sections of the ecotoxicological model organism *E. fetida* combining FTIR and MS imaging techniques. Each step within this workflow is optimized to satisfy the demands of the implementation of both methods on a single sample for a sequential analysis (Fig. 1). First, for the preparation of sample sections suitable for analysis, we used 3% CMC as embedding medium to ensure tissue integrity. CMC is compatible with both measurement techniques, since it facilitates preparation of tissue sections which is a prerequisite for FTIR imaging and does not interfere with MS imaging analysis (Goodwin et al. 2012). Because sample thickness is a limiting factor for FTIR analysis (when data are acquired in transmission mode; reviewed in Tuck et al. 2021), we made 20 µm-thick sections.

### FTIR imaging and data analysis

**Fig. 2:**
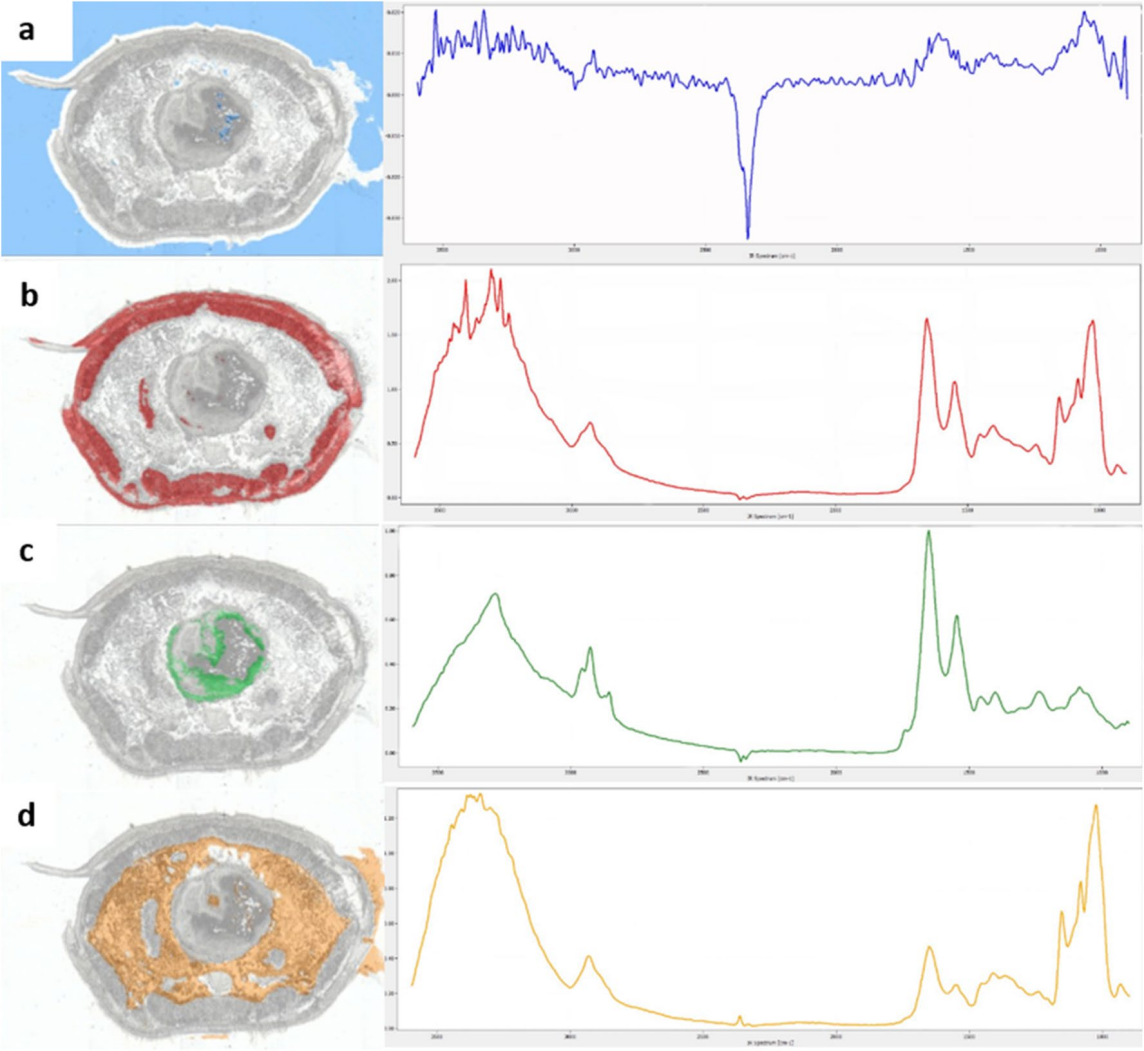
FTIR data analysis via random decision forest classification of a tissue section of *E. fetida*. **a** Result of the RDF application for class 1 “background” as overlay over the optical image of the section and an exemplary IR spectrum representing this class; this class is represented with blue coloration. **b** Result of the RDF application for class 2 “body wall” as overlay over the optical image of the section and an exemplary IR spectrum representing this class; this class is represented with red coloration. **c** Result of the RDF application for class 3 “digestive system” as overlay over the optical image of the section and an exemplary IR spectrum representing this class; this class is represented with green coloration. **d** Result of the RDF application for class 4 “other tissue” as overlay over the optical image of the section and an exemplary IR spectrum representing this class; this class is represented with orange coloration

The first step of the analytical workflow was FTIR imaging. This method was applied first, as it delivers a rapid, noninvasive chemical overview of the analyzed section. Further, it makes it possible to define regions of interest within a tissue section for the subsequent analysis via MS imaging, which is not possible using classical histological staining such as H&E. Considering the anatomy of earthworms, there are two main tissue areas that first come into contact with possible pollutants and therefore are of special interest for further studies focusing on toxicology. These tissue areas are (1) the body wall, which includes the cuticle, the epidermis and the circular and longitudinal muscles, building a functional unit in the Annelida, constituting the outer body barrier to the environment, and (2) the digestive system, which is in contact with ingested food and possible contaminants (Bilej et al. 2010). We applied a machine learning algorithm based on RDF on the FTIR data with the goal of visualizing these different tissue areas within the sections. RDF models have been proven capable of separating different groups within intricate data samples (Horning 2010; Mayerich et al. 2014). We defined two main tissue classes, “body wall” and “digestive system,” and two additional classes, defined as “other tissue” and “background”. The RDF model assigns the class according to similarities or dissimilarities in the IR spectra of biological tissue, which are usually in the fingerprint region around 900–1450 cm^−1^ (lipids, carbohydrates and nucleic acids, also contribution from proteins), 1500–1700 cm^−1^ (amide I and II region corresponding to proteins) and 2500–3500 cm^−1^ (mainly lipids) (Coates 2006; Movasaghi et al. 2008; Baker et al. 2014). The RDF was validated by Monte Carlo cross-validation (Xu and Liang 2001; Dubitzky et al. 2007). The results of the validation are presented as the sensitivity, specificity and precision with which the model correctly assigns a data point to the respective class (details on this approach can be found in Table S1, Fig. S1). The results of the classification model with an exemplary IR spectrum are displayed in Fig. 2. The different tissue types/classes (classes: 1 = background [blue], Fig. 2a; 2 = body wall [red], Fig. 2b; 3 = digestive system [green], Fig. 2c; 4= other tissue [orange], Fig. 2d) can be clearly differentiated based on the spectral data.

To prove the reproducibility of the model, we applied it on five different sections of three different individuals (worms), resulting in the same correlation with the respective tissue types (Fig. S1). The RDF enables rapid tissue type characterization (measurement of one section ~ 20 min; application of our RDF model ~ 5–10 min). The relatively fast data acquisition technique has the advantage over common histopathology that fewer steps such as fixation or staining are needed (Tian et al. 2015). In addition, the application of an RDF model for data analysis reduces observer bias which might occur if tissue is analyzed solely by optical microscopic observation (Tian et al. 2015). In concordance with our study, Li et al. (2005) already showed that FTIR spectroscopy is suitable for the diagnosis and differentiation of healthy tissue, inflammation (gastritis) and malignancy (gastric cancer) in biopsies, using another algorithm that can be trained (supervised linear discriminant analysis) for multiple group classifications. In our study we used classification based on random decision forests, which is said to be one of the most powerful machine learning algorithms (Rana et al. 2020). The advantage of the RDF used in our study is that RDF selects a random subset of features that enables increased variation among the trees constituting the model. This results in a higher percentage of accuracy in classification taking into account the low correlation across trees (Nurwulan and Selamaj 2020). In addition, RDF algorithms are highly capable in dealing with complex data systems (Santana et al. 2018), which in combination with cross-validation offers a promising tool for the targeted (e.g. pollutant detection) data analysis of FTIR images (Hufnagl et al. 2019).

For future applications, one could include further RDF classes of tissues, or implement and extend RDF-based models with new classes for the detection of specific water-soluble or particulate pollutants, as recently shown in environmental samples (Hufnagl et al. 2019). This might be promising for ecotoxicology, as it has already been shown that vibrational spectroscopy can be used for nondestructive and label-free tissue analysis (for detailed review refer to Prentice et al. 2017) and even for analysis of drug penetration within tissue, for example (Mendelsohn et al. 2003; Xiao et al. 2005; Jiang et al. 2008). As an example, Mendelsohn et al. (2003) used IR spectroscopy for the analysis of the penetration of dimethyl sulfoxide (DMSO) and propylene glycol into skin.

Overall, data analysis of the FTIR spectra via RDF classification facilitates the identification of regions of interest in tissues which can then be further analyzed with a higher spatial and molecular resolution using a MS imaging approach.

### MS imaging and data analysis

**Fig. 3:**
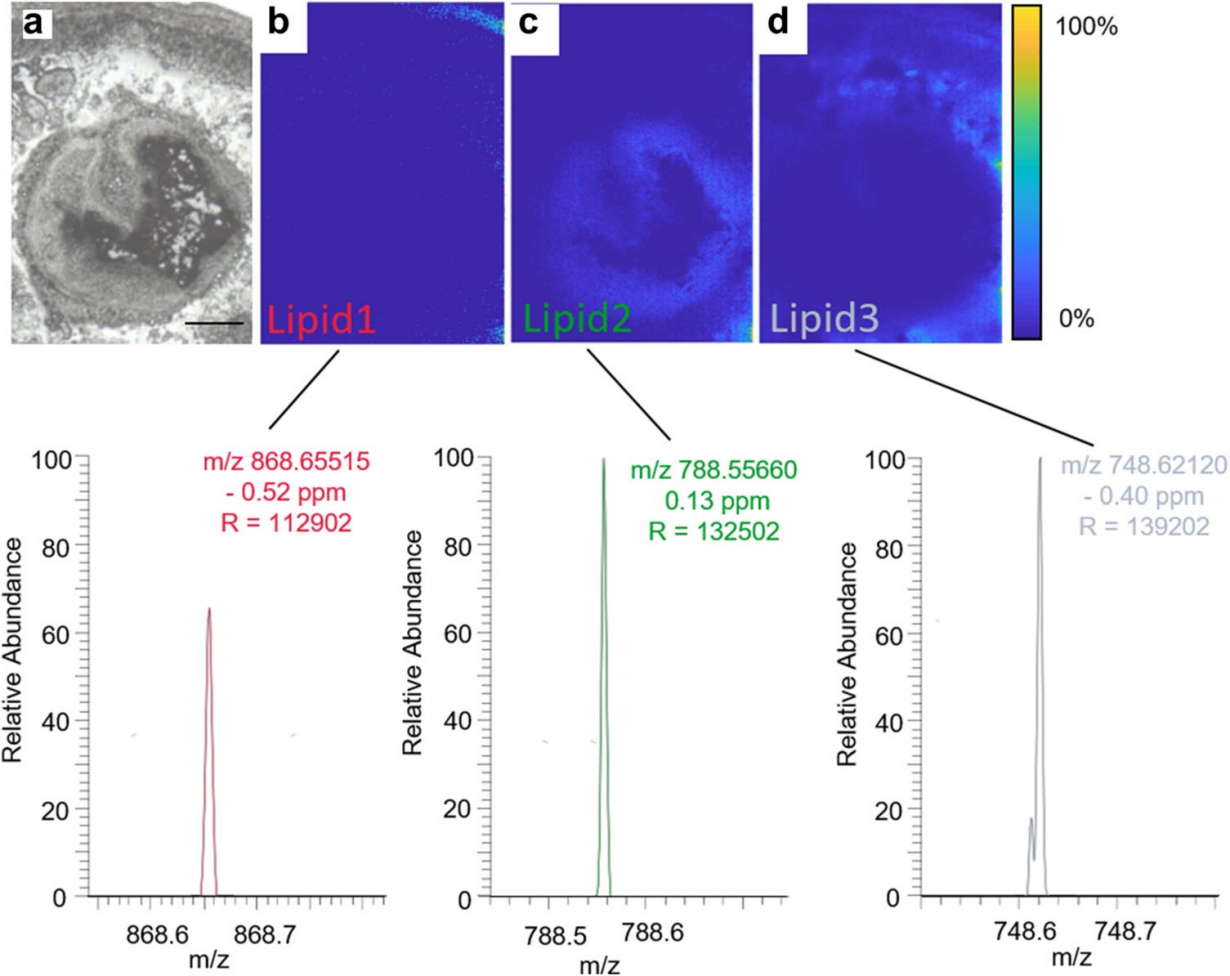
Single ion images displaying different tissue areas in the analyzed *E. fetida* tissue section. **a** Optical image demonstrating the region of interest measured by MS imaging. **b** Distribution of PC (O-40:1) [M+K]+ displaying the epidermis of *E. fetida*. **c** Distribution of PC (O-36:5) [M+Na]+ displaying the distribution of this lipid in the digestive system of *E. fetida*. **d** Distribution of PC (O-34:0) [M+H]+ displaying the distribution of this lipid in the area of the coelomic fluid and muscle tissue. Scale bar = 250 µm

A high-resolution analysis step using MALDI-MSI was applied to assign specific lipids to target tissues (e.g., digestive tissue) identified with FTIR analysis. Figure 3a shows the region of interest (ROI; target tissue) within the optical image of the tissue section. The outermost part of the tissue section could be partly visualized by the distribution of lipid 1 (Fig. 3b). This lipid could be tentatively identified by accurate mass, confirmed by MS/MS (see Fig. S2) as phosphatidylcholine (PC-O-40:1) [M+K]^+^ (*m*/*z* 868.6556; RMSE: 1.2810 ppm, 1103 spectra). “Lipid 2” was tentatively identified as PC (O-36:5) [M+Na]^+^ (*m*/*z* 788.5565; RMSE: 0.6530 ppm, 30,824 spectra) and shows a distribution with high intensity in the digestive tissue (Fig. 3c). The tissue region between the outermost part of the body wall and the digestive tissue could be visualized by the distribution of lipid 3. This could be tentatively identified by accurate mass, confirmed by MS/MS as PC (O-34:0) [M+H]^+^ (*m*/*z* 748.6215; RMSE 0.5396 ppm, 55,232 spectra) (Fig. 3d). The distribution of the lipids, and thereby the reproducibility of the measurements, was demonstrated in total in five different sections of two different individuals (worms) (Fig. S3).

Here we focused on the lipid distribution within different tissue parts of *E. fetida*, as lipids are the main components of the tissue architecture and take part in the organisms’ function (Sparvero et al. 2012). Further, it is already known that *E. fetida* shows an altered lipid metabolism when exposed to toxicants. For instance, Mayilswami et al. (2017) were able to show, by analysis of the transcriptome, that the lipid metabolism of *E. fetida* is impacted by pollutants such as benzo[a]pyrene. The lipid signatures can be used as a biological marker to detect physiological changes in tissue, as shown by Barbacci et al. (2017). Hence, this approach could be used to study the effects of environmental pollutants at the tissue level. Another aspect that might be of interest in studying the effects of pollutants is that the oxidation of molecules such as lipids can be found as a reaction to environmental stress in earthworms (reviewed in Solé 2020). Lipid oxidation in relation to a stress reaction in *Eisenia* is commonly studied by analysis of malondialdehyde, the final product of lipid peroxidation, as marker for oxidative damage (Zhang et al. 2013; Song et al. 2018). Such an analysis usually involves the homogenization of tissue samples for the extraction and analysis of an analyte (Zhang et al. 2013; Zhou et al. 2013; Shao et al. 2018). In contrast, MS imaging allows for label-free on-tissue lipid analysis and in addition enables the analysis of oxidative changes in lipids (Desbenoit et al. 2018). This allows us to extend the evaluation of lipid modifications with spatial information, which can also be linked to the underlying biological processes which are affected.

### Multimodal imaging approach

**Fig. 4:**
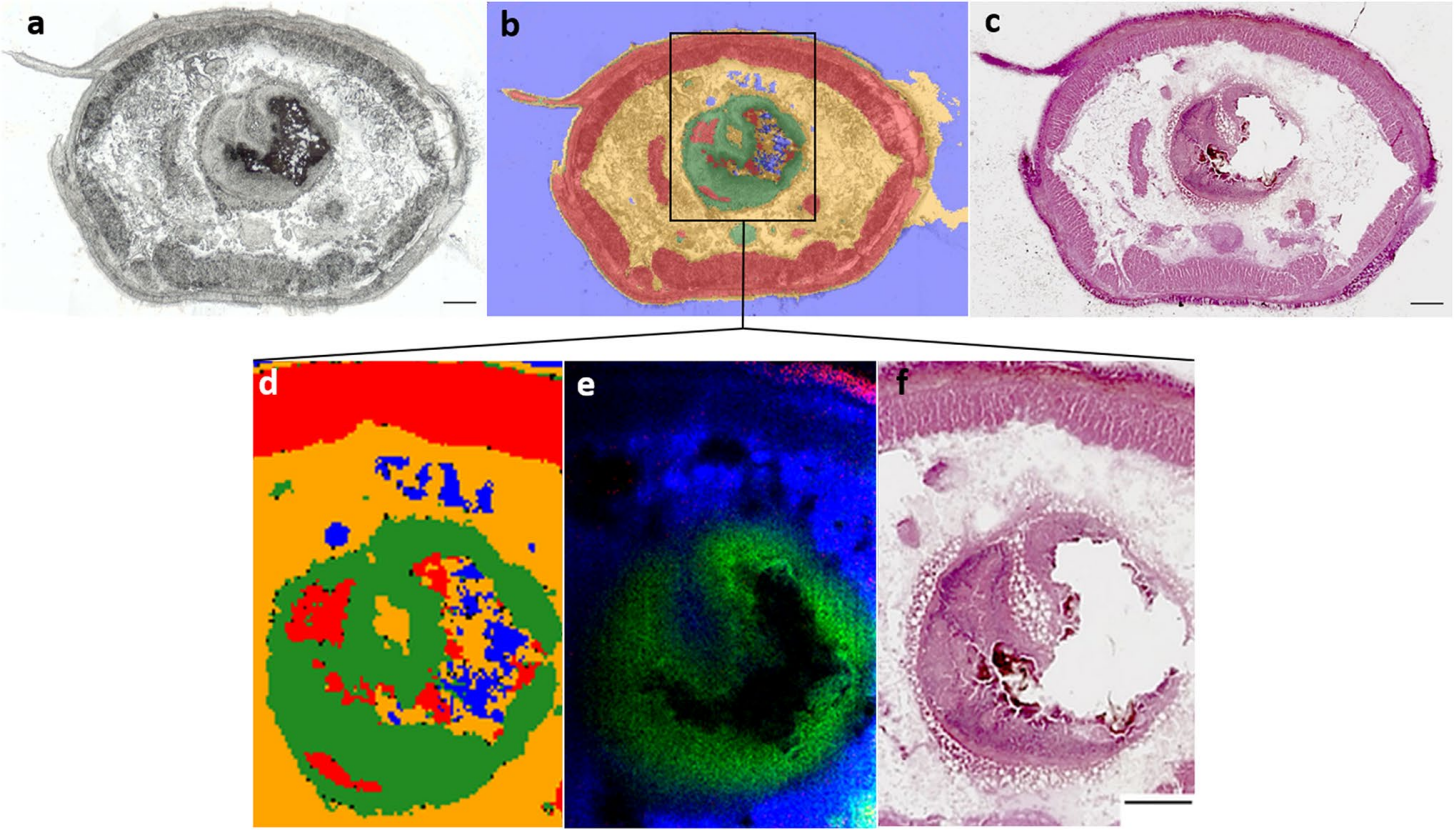
Result of the multimodal imaging approach. **a** Bright-field microscopic image of the analyzed tissue section of *E. fetida;* scale bar = 250 µm. **b** Overlay of the application of the RDF model as data analysis of the FTIR data and the bright-field microscopic image. **c** H&E staining of the section after FTIR and MALDI-MSI application; scale bar = 250 µm. **d** Results of the RDF model application of the FTIR data; zoomed in on the region imaged by MALDI-MSI, colors are according to the results of the RDF analysis. **e** Results of the MALDI-MSI analysis of the region of interest; the identified lipids are colored in red, blue and green; **f** H&E-stained section; zoomed in on the region analyzed by MALDI-MSI. Scale bars = 250 µm

Figure 4 shows the results obtained of the sequential workflow together with the optional H&E staining of the same tissue section which was used to evaluate the correlation of FTIR and MS imaging results with histological features. The sequence as shown in Fig. 1 was followed, since, for example, staining with H&E beforehand would make a measurement with MS imaging impossible, as the mandatory treatment of the section with ethanol and isopropanol extracts lipids. Hence, FTIR imaging, which ensures tissue integrity while still enabling us to define regions of interest for subsequent MS imaging, was used in our workflow. The image obtained by applying the RDF model is shown as an overlay over the optical image in Fig. 4a, b. The tissue classes (background, body wall, digestive system and other tissue) could be differentiated based on the spectral data corresponding to each tissue type. In comparison to the H&E-stained image (Fig. 4c, f), the class in red (“body wall”) matches with the cuticle, the epidermis and the circular and longitudinal muscles. The body wall was further differentiated by lipids identified in the MS imaging experiments, (red; Fig. 4e; corresponding to single ion image Fig. 3b). The comparison to the H&E-stained image shows that the lipid colored in red corresponds to the epidermis of the earthworm (Fig. 4e, f). The RDF class referring to the digestive system matches the distribution of lipid 2 (Fig. 4d, e).

The multimodal workflow enables rapid analysis of the FTIR imaging data by supervised machine learning algorithms such as the RDF, and its results can be used to facilitate the determination of regions of interest for more detailed measurements using MS imaging. Our proposed workflow enables a targeted analysis of potential effects of pollutants in *E. fetida*. This is supported by the fact that multimodal imaging using FTIR and MS imaging is already applied in medical research. For instance, Rabe et al. (2018) used FTIR and MS imaging for tumor localization in thin sections of mouse brain and were able to detect disease-specific lipid patterns. Hence, FTIR data analysis using algorithms like RDF open up the possibility for automated, time-saving, high-throughput tissue characterization and classification of specific regions of the tissue. Consequently, the combination of the two methods for an established model organism in ecotoxicological research represents an improved approach for investigating the effects of pollutants at the tissue level.

## Conclusions

Our multimodal workflow enables the consecutive analysis of sections of non-preserved specimen of the ecotoxicological model organism *E. fetida*. FTIR-analysis with subsequent RDF application allows the quick classification of different tissue types within complex biological cross sections. It further excludes observer bias and enables a quick identification of regions of interest for subsequent analysis. To this, MS imaging adds molecular information by correlating specific lipids with the different tissue types. These lipids could be used to detect spatially resolved physiological modifications which are induced by environmental contaminants. Our workflow provides a first step to a time-saving tool for ecotoxicological research by offering a quick, non-invasive multimodal analysis of tissues or cross-sections of *E. fetida*. Therefore, our approach has the potential to improve toxicology targeting effects at the tissue level on terrestrial biota.

## Supplementary Information

Below is the link to the electronic supplementary material.

Supplementary file1 (PDF 1231 KB)

## Data Availability

The data that support the findings of this study are available from the corresponding author upon reasonable request.

## References

[CR1] Ali S, Awan Z, Mumtaz S (2020). Cardiac toxicity of heavy metals (cadmium and mercury) and pharmacological intervention by vitamin C in rabbits. Environ Sci Pollut Res.

[CR2] Alves A, Weis GCC, Unfer TC (2019). Caffeinated beverages contribute to a more efficient inflammatory response: evidence from human and earthworm immune cells. Food Chem Toxicol.

[CR3] Antora SA, Hossain MN, Rahman MM (2019). Detection of adulteration in edible oil using FT-IR spectroscopy and machine learning. Int J Biochem Res Rev.

[CR4] Baker MJ, Trevisan J, Bassan P (2014). Using Fourier transform IR spectroscopy to analyze biological materials. Nat Protoc.

[CR5] Ballabio D, Grisoni F, Todeschini R (2018). Multivariate comparison of classification performance measures. Chemom Intell Lab Syst.

[CR6] Barbacci DC, Roux A, Muller L (2017). Mass spectrometric imaging of ceramide biomarkers tracks therapeutic response in traumatic brain injury. ACS Chem Neurosci.

[CR7] Belsky J, Joshi NK (2020). Effects of fungicide and herbicide chemical exposure on apis and non-apis bees in agricultural landscape. Front Environ Sci.

[CR8] Bilej M, Procházková P, Šilerová M, Josková R, Söderhäll K (2010). Earthworm immunity. Invertebrate immunity. Advances in experimental medicine and biology.

[CR9] Breiman L (2001). Random forests. Mach Learn.

[CR10] Cazares LH, Troyer D, Mendrinos S (2009). Imaging mass spectrometry of a specific fragment of mitogen-activated protein kinase/extracellular signal-regulated kinase kinase kinase 2 discriminates cancer from uninvolved prostate tissue. Clin Cancer Res.

[CR11] Chen R, Hui L, Sturm RM, Li L (2009). Three dimensional mapping of neuropeptides and lipids in crustacean brain by mass spectral imaging. J Am Soc Mass Spectrom.

[CR12] Coates J (2006). Interpretation of infrared spectra, a practical approach. Encycl Anal Chem.

[CR13] Cornett DS, Reyzer ML, Chaurand P, Caprioli RM (2007). MALDI imaging mass spectrometry: molecular snapshots of biochemical systems. Nat Methods.

[CR14] De SFB, Mazivila SJ, Gontijo LC, Neto WB (2018). Rapid discrimination between authentic and adulterated andiroba oil Using FTIR-HATR ): spectroscopy and random forest. Food Anal Methods.

[CR15] Dempster WT (1963). The mechanics of paraffin sectioning by the microtome. Stain Technol.

[CR16] Desbenoit N, Walch A, Spengler B (2018). Correlative mass spectrometry imaging, applying time-of-flight secondary ion mass spectrometry and atmospheric pressure matrix-assisted laser desorption/ionization to a single tissue section. Rapid Commun Mass Spectrom.

[CR17] Diem M (2015) Modern vibrational spectroscopy and micro-spectroscopy: theory, instrumentation and biomedical applications. Wiley

[CR18] Dowhan W, Bogdanov M (2002). Functional roles of lipids in membranes. In new comprehensive biochemistry.

[CR19] Dubitzky W, Granzow M, Berrar DP (2007). Fundamentals of data mining in genomics and proteomics.

[CR20] Giorgini E, Randazzo B, Gioacchini G (2018). New insights on the macromolecular building of rainbow trout (*O. mykiss*) intestine: FTIR Imaging and histological correlative study. Aquaculture.

[CR21] Goodwin RJA, Nilsson A, Borg D (2012). Conductive carbon tape used for support and mounting of both whole animal and fragile heat-treated tissue sections for MALDI MS imaging and quantitation. J Proteomics.

[CR22] Großerueschkamp F, Kallenbach-Thieltges A, Behrens T (2015). Marker-free automated histopathological annotation of lung tumour subtypes by FTIR imaging. Analyst.

[CR23] Horning N (2010). Random Forests: An algorithm for image classification and generation of continuous fields data sets. Int Conf Geoinform Spat Infrastruct Dev Earth Allied Sci.

[CR24] Huber K, Khamehgir-Silz P, Schramm T (2018). Approaching cellular resolution and reliable identification in mass spectrometry imaging of tryptic peptides. Anal Bioanal Chem.

[CR25] Hufnagl B, Steiner D, Renner E (2019). A methodology for the fast identification and monitoring of microplastics in environmental samples using random decision forest classifiers. Anal Methods.

[CR26] Jiang J, Boese M, Turner P, Wang RK (2008). Penetration kinetics of dimethyl sulphoxide and glycerol in dynamic optical clearing of porcine skin tissue in vitro studied by Fourier transform infrared spectroscopic imaging. J Biomed Opt.

[CR27] Jiang X, Chang Y, Zhang T (2020). Toxicological effects of polystyrene microplastics on earthworm (*Eisenia fetida*). Environ Pollut.

[CR28] Kedzierski M, Falcou-Préfol M, Kerros ME (2019). A machine learning algorithm for high throughput identification of FTIR spectra: application on microplastics collected in the mediterranean sea. Chemosphere.

[CR29] Khalil SM, Römpp A, Pretzel J (2015). Phospholipid topography of whole-body sections of the anopheles stephensi mosquito, characterized by high-resolution atmospheric-pressure scanning microprobe matrix-assisted laser desorption/ionization mass spectrometry imaging. Anal Chem.

[CR30] Kimber JA, Foreman L, Turner B (2016). FTIR spectroscopic imaging and mapping with correcting lenses for studies of biological cells and tissues. Faraday Discuss.

[CR31] Lapied E, Nahmani JY, Moudilou E (2011). Ecotoxicological effects of an aged TiO2 nanocomposite measured as apoptosis in the anecic earthworm Lumbricus terrestris after exposure through water, food and soil. Environ Int.

[CR32] Leblanc GA (2004). Basics of environmental toxicology.

[CR33] Li QB, Sun XJ, Xu YZ (2005). Diagnosis of gastric inflammation and malignancy in endoscopic biopsies based on fourier transform infrared spectroscopy. Clin Chem.

[CR34] Li M, Ma X, Saleem M (2020). Biochemical response, histopathological change and DNA damage in earthworm (*Eisenia fetida*) exposed to sulfentrazone herbicide. Ecol Indic.

[CR35] Liebeke M, Strittmatter N, Fearn S (2015). Unique metabolites protect earthworms against plant polyphenols. Nat Commun.

[CR36] Liu W, Nie H, Liang D (2020). Phospholipid imaging of zebrafish exposed to fipronil using atmospheric pressure matrix-assisted laser desorption ionization mass spectrometry. Talanta.

[CR37] Mayerich DM, Walsh M, Kadjacsy-Balla A (2014). Breast histopathology using random decision forests-based classification of infrared spectroscopic imaging data. Med Imaging Digit Pathol.

[CR38] Mayilswami S, Krishnan K, Naidu R, Megharaj M (2017). Transcriptome analysis of Eisenia fetida chronically exposed to benzo(a)pyrene. Environ Technol Innov.

[CR39] Mendelsohn R, Chen H-C, Rerek ME, Moore DJ (2003). Infrared microspectroscopic imaging maps the spatial distribution of exogenous molecules in skin. J Biomed Opt.

[CR40] Molnár L, Engelmann P, Somogyi I (2012). Cold-stress induced formation of calcium and phosphorous rich chloragocyte granules (chloragosomes) in the earthworm *Eisenia fetida*. Comp Biochem Physiol A Mol Integr Physiol.

[CR41] Movasaghi Z, Rehman S, Rehman IU (2008). Fourier transform infrared (FTIR) spectroscopy of biological tissues. Appl Spectrosc Rev.

[CR42] Nagel R (2002). DarT: the embryo test with the Zebrafish Danio rerio–a general model in ecotoxicology and toxicology. Altex.

[CR43] Nayak S, Mishra CSK, Guru BC, Samal S (2018). Histological anomalies and alterations in enzyme activities of the earthworm Glyphidrillus tuberosus exposed to high concentrations of phosphogypsum. Environ Monit Assess.

[CR44] Nguyen MH, Zhang Y, Wang F (2021). Machine learning to extract physiological parameters from multispectral diffuse reflectance spectroscopy. J Biomed Opt.

[CR45] Niehoff A-C, Kettling H, Pirkl A (2014). Analysis of *Drosophila* Lipids by matrix-assisted laser Desorption/Ionization mass spectrometric imaging. Anal Chem.

[CR46] Nurwulan NR, Selamaj G (2020). Random forest for human daily activity recognition. J Phys Conf Ser.

[CR47] Prentice BM, Caprioli RM, Vuiblet V (2017). Label-free molecular imaging of the kidney. Kidney Int.

[CR48] Rabe JH, Sammour DA, Schulz S (2018). Fourier transform infrared microscopy enables guidance of automated mass spectrometry imaging to predefined tissue morphologies. Sci Rep.

[CR49] Race AM, Styles IB, Bunch J (2012). Inclusive sharing of mass spectrometry imaging data requires a converter for all. J Proteomics.

[CR50] Rana D, Jena SP, Pradhan SK (2020). Performance comparison of PCA and LDA with linear regression and random forest for iris flower classification. PalArch’s J Archaeol Egypt/egyptol.

[CR51] Rodrigues LM, Carvalho LFS, Bonnier F (2018). Evaluation of inflammatory processes by FTIR spectroscopy. J Med Eng Technol.

[CR52] Rodriguez-Seijo A, Lourenço J, Rocha-Santos TAP (2017). Histopathological and molecular effects of microplastics in Eisenia andrei Bouché. Environ Pollut.

[CR53] Römpp A, Guenther S, Schober Y (2010). Histology by mass spectrometry: label-free tissue characterization obtained from high-accuracy bioanalytical imaging. Angew Chemie Int Ed.

[CR54] Sanderson C, Emmanuel J, Emmanual J, Campbell P (1988). A historical review of paraffin and its development as an embedding medium. J Histotechnol.

[CR55] Schober Y, Guenther S, Spengler B, Römpp A (2012). Single cell matrix-assisted laser desorption/ionization mass spectrometry imaging. Anal Chem.

[CR56] Shao Y, Wang J, Du Z (2018). Toxic effect of [Omim]BF4 and [Omim]Br on antioxidant stress and oxidative damage in earthworms (*Eisenia fetida*). Environ Toxicol Pharmacol.

[CR57] Solé M (2020). Biomarkers in earthworms. In: the handbook of environmental chemistry.

[CR58] Song P, Ping L, Gao J (2018). Ecotoxicological effects of fertilizers made from pulping waste liquor on earthworm Eisenia fetida. Ecotoxicol Environ Saf.

[CR59] Sparvero LJ, Amoscato AA, Dixon CE (2012). Mapping of phospholipids by MALDI imaging (MALDI-MSI): Realities and expectations. Chem Phys Lipids.

[CR60] Spurgeon DJ, Weeks JM, Van Gestel CAM (2003). A summary of eleven years progress in earthworm ecotoxicology. Pedobiologia (jena).

[CR61] Steven RT, Race AM, Bunch J (2013). para-Nitroaniline is a promising matrix for MALDI-MS imaging on intermediate pressure MS systems. Am Soc Mass Spectrom.

[CR62] Sud M, Fahy E, Cotter D (2007). LMSD: LIPID MAPS structure database. Nucleic Acids Res.

[CR63] Tian P, Zhang W, Zhao H (2015). Intraoperative diagnosis of benign and malignant breast tissues by fourier transform infrared spectroscopy and support vector machine classification. Int J Clin Exp Med.

[CR64] Tirado-Ballestas I, Caballero-Gallardo K, Olivero-Verbel J (2020). Toxicological effects of bituminous coal dust on the earthworm *Eisenia fetida* (*Oligochaeta*: *Lumbricidae*). Ecotoxicology.

[CR65] Tuck M, Blanc L, Touti R (2021). Multimodal imaging based on vibrational spectroscopies and mass spectrometry imaging applied to biological tissue : a multiscale and multi-omics review. Pre-Anal Factors.

[CR66] Wang K, Pang S, Mu X (2015). Biological response of earthworm, *Eisenia fetida*, to five neonicotinoid insecticides. Chemosphere.

[CR67] Welte MA, Gould AP (2017). Lipid droplet functions beyond energy storage. Biochim Biophys Acta Mol Cell Biol Lipids.

[CR68] Westad F, Marini F (2015). Validation of chemometric models - A tutorial. Anal Chim Acta.

[CR69] Wood BR, Bambery KR, Evans CJ (2006). A three-dimensional multivariate image processing technique for the analysis of FTIR spectroscopic images of multiple tissue sections. BMC Med Imaging.

[CR70] Xiao C, Moore DJ, Flach CR, Mendelsohn R (2005). Permeation of dimyristoylphosphatidylcholine into skin - Structural and spatial information from IR and Raman microscopic imaging. Vib Spectrosc.

[CR71] Xu QS, Liang YZ (2001). Monte Carlo cross validation. Chemom Intell Lab Syst.

[CR72] Zhang Q, Zhu L, Wang J (2013). Oxidative stress and lipid peroxidation in the earthworm *Eisenia fetida* induced by low doses of fomesafen. Environ Sci Pollut Res.

[CR73] Zhang Y, Qin L, Sun J (2020). Metabolite changes associated with earthworms (*Eisenia fetida*) graphene exposure revealed by matrix-assisted laser desorption/ionization mass spectrometry imaging. Ecotoxicol Environ Saf.

[CR74] Zhao C, Xie P, Yong T (2018). MALDI-MS imaging reveals asymmetric spatial distribution of lipid metabolites from bisphenol S-induced nephrotoxicity. Anal Chem.

[CR75] Zhou CF, Wang YJ, Li CC (2013). Subacute toxicity of copper and glyphosate and their interaction to earthworm (*Eisenia fetida*). Environ Pollut.

