# Supplementary material for: Classification of target tissues of Eisenia fetida using sequential multimodal chemical analysis and machine learning
Source: Histochem Cell Biol. 2021 Nov 8;157(2):127–37. doi: 10.1007/s00418-021-02037-1 (PMC8847259; doi:10.1007/s00418-021-02037-1)
Supplement: Supplementary file 1 — Supplementary file1 (PDF 1231 KB) [file 418_2021_2037_MOESM1_ESM.pdf]

Supplemental Material to the Manuscript

Sequential analysis of *Eisenia fetida* tissue sections: Classification of target tissue using multimodal chemical analysis and machine learning

Sven Ritschar[1đ], Elisabeth Schirmer[2đ], Benedikt Hufnagl[3,4], Martin G. J. Löder[1], Andreas Römpp[2*] and Christian Laforsch[1*]

[1]Department of Animal Ecology I and BayCEER, University of Bayreuth, Germany

[2]Department of Bioanalytical Sciences and Food analysis, University of Bayreuth, Germany

[3]Institute of Chemical Technologies and Analytics, TU Vienna, Austria

[4]Purency GmbH, Walfischgasse 8/34, AT-1010 Vienna, Austria

đcontributed equally to this work

Corresponding author*:

Christian Laforsch, Christian.Laforsch@uni-bayreuth.de

Andreas Römpp, Andreas.Roempp@uni-bayreuth.de

Table S1: Results of the Monte-Carlo cross validation, calculated for each class and overall classification performance measures of the model based on random decision forest classification.

**CLASS**	**1 (BACKGROUND)**	**2 (MUSCLE TISSUE)**	**3 (DIGESTIVE TISSUE)**	**4 (OTHER TISSUE)**
SENSITIVITY	1	0.9833	0.9167	0.8167
SPECIFICITY	0.9956	0.9678	0.9622	0.9800
PRECISION	0.9868	0.9105	0.8900	0.9316

**OVERALL CLASSIFICATION PERFORMANCE MEASURES**	
TOTAL ACCURACY	0.9292
COHEN'S KAPPA	0.9056
EMCC	0.9064

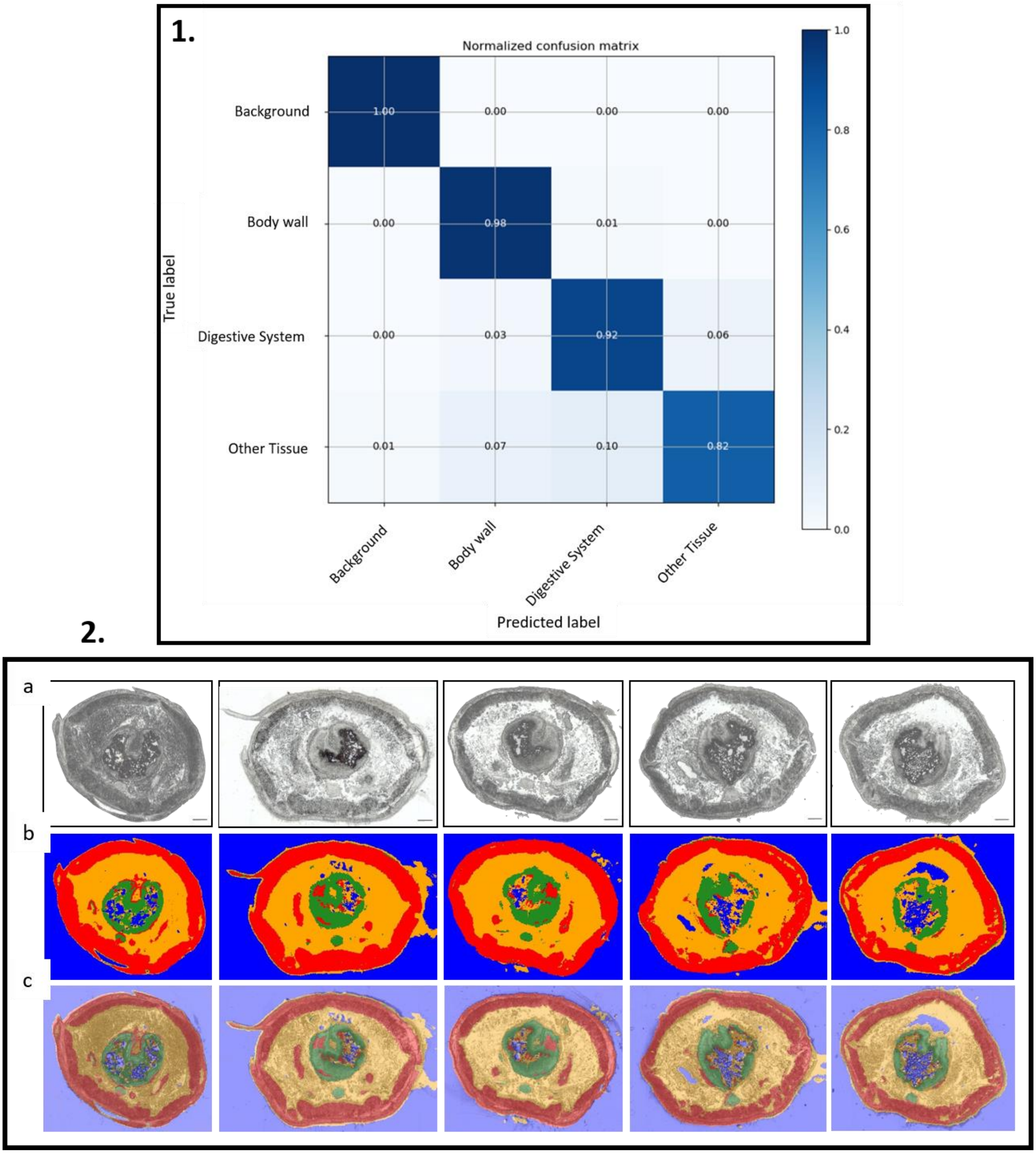

Figure S1: Results of the cross-validation of the RDF by Monte-Carlo- Cross validation and replication of the application of the RDF model on five individual tissue sections of *E. fetida*. **1.** Confusion matrix, the entries of the main diagonal represent the correctly classified cases whereas the off-diagonal elements are the wrongly classified cases. **2. a:** optical image of the respective thin-section of *E. fetida*. **b:** Result of the application of the RDF on the FTIR-hyperspectral images of the respective section of *E. fetida*, the classes have been assigned their specific color (blue=" Background", red=" Muscle", green=" Digestive system" and orange =" Other tissue"). **c:** Overlay of the results of the RDF (b) and the optical images (a). Scale bar= 250µm.

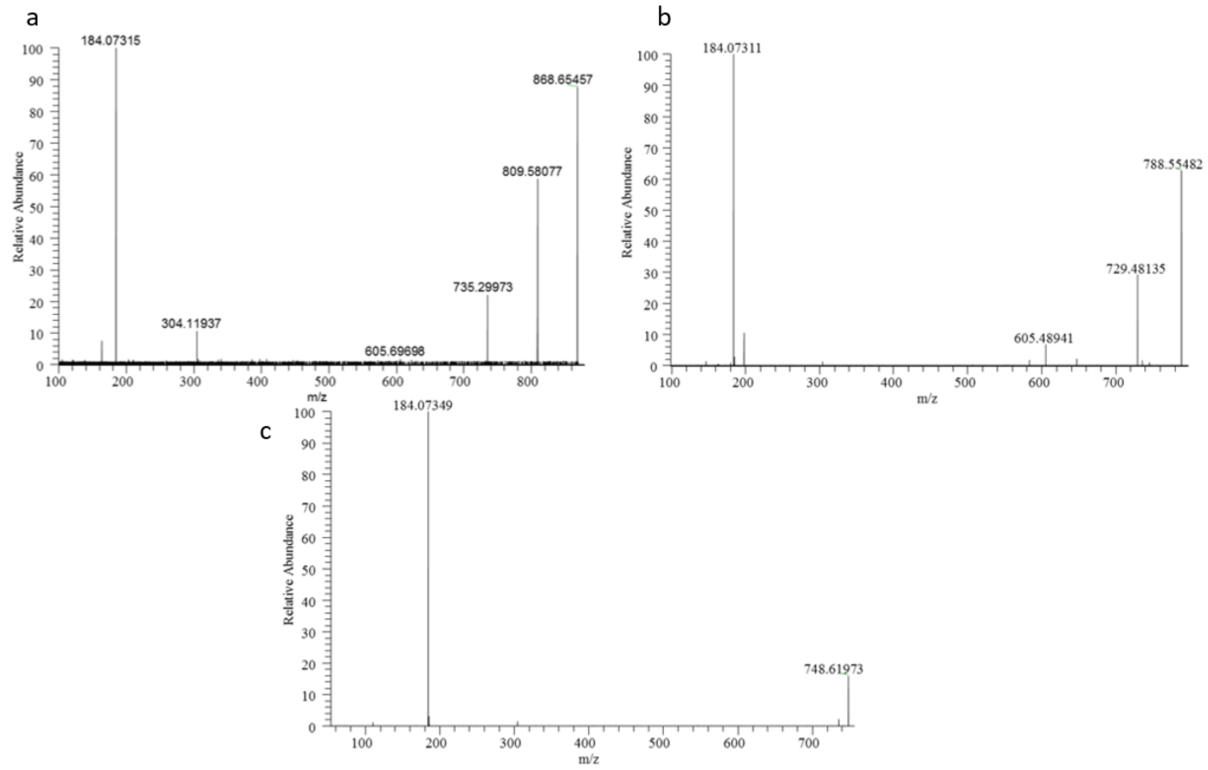

Figure S2: MS/MS of the tentatively identified lipids in cross sections of *E. fetida*. **a:** Lipid 1, PC (O-40:1) $[M+K]^+$ (m/z 868.6556; RMSE: 1.2810 ppm, 1103 spectra) identified in the epidermis of the cross section of *E. fetida*; **b:** Lipid 2, PC (O-36:5) $[M+Na]^+$ (m/z 788.5565; RMSE: 0.6530 ppm, 30824 spectra),identified in the digestive system of *E. fetida*; **c:** Lipid 3, PC (O-34:0) $[M+H]^+$ (m/z 748.6215; RMSE 0.5396 ppm, 55232 spectra) identified in the coelom of *E. fetida*.

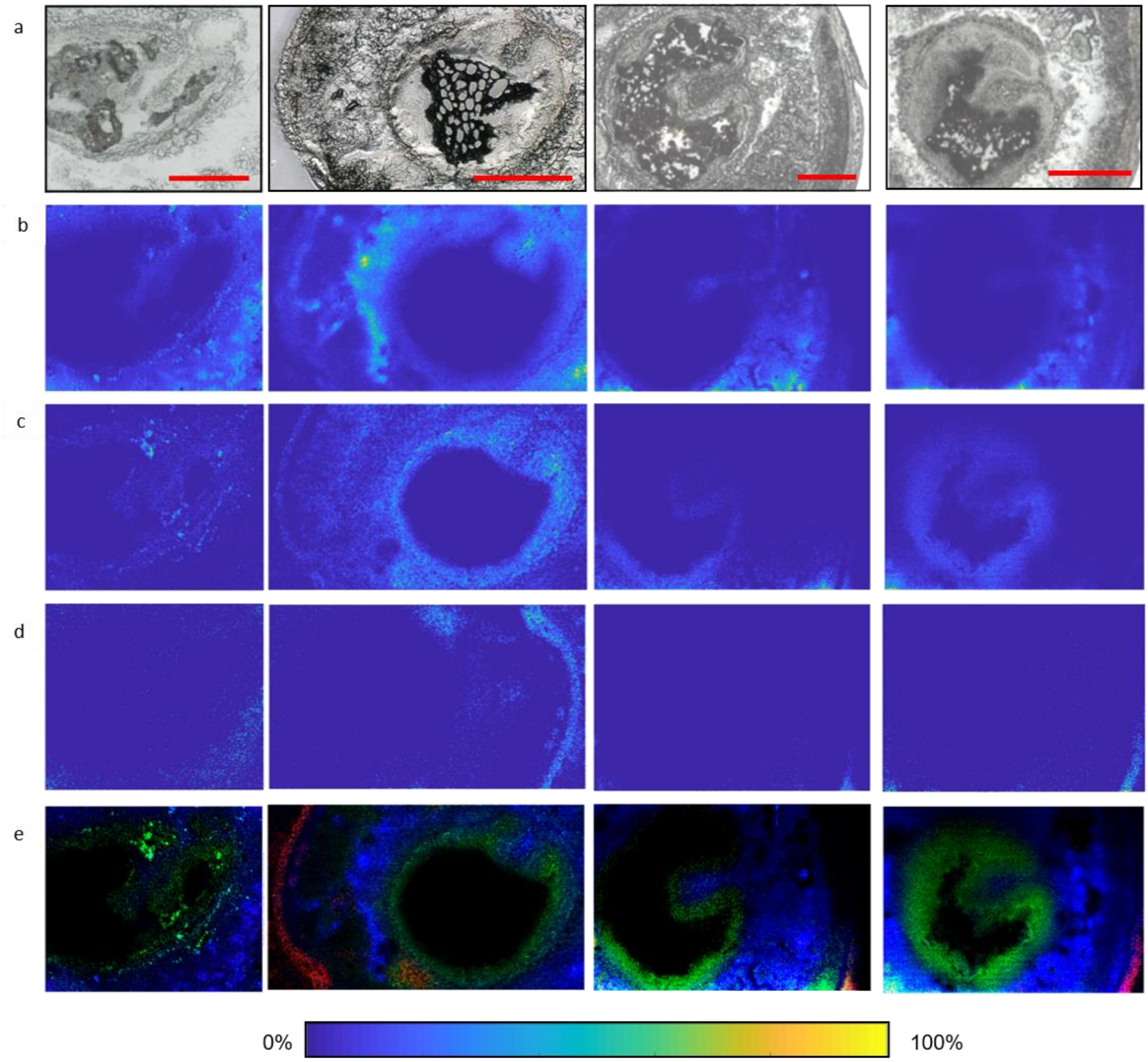

Figure S3: Replication of the MALDI-MSI data evaluation on four individual tissue sections of *E. fetida*. **a:** optical image of the areas selected for MALDI-MSI measurements. **b:** Distribution of PC (O-34:0) [M+H]+ (m/z= 748.62120). **c:** Distribution of the tentatively identified lipid PC (O-36:5) [M+Na]+ (m/z= 788.55660). **d:** Distribution of the tentatively identified lipid PC (O-40:1) [M+K]+ (m/z= 868.65515). **e:** RGB overlay of the three identified lipids with their respective colors (b= blue, c= green and d= red). Scale bar= 500µm.
